# Incorporating oxygenation levels in analytical DNA-damage models—quantifying the oxygen fixation mechanism

**DOI:** 10.1088/1361-6560/ac0b80

**Published:** 2021-07-09

**Authors:** Frank Van den Heuvel, Anna Vella, Francesca Fiorini, Mark Brooke, Mark A Hill, Tim Maughan

**Affiliations:** 1 University of Oxford, Department of Oncology, Oxford, United Kingdom; 2 Zuidwest Radiotherapeutic Institute, Vlissingen, Zeeland, The Netherlands; 3 Oxford University Hospitals, Department of Hæmatology & Oncology, Oxford, United Kingdom; 4 Rutherford Cancer Centre Thames Valley, Reading, United Kingdom

**Keywords:** oxygen, radiation biology, treatment planning

## Abstract

*Purpose.* To develop a framework to include oxygenation effects in radiation therapy treatment planning which is valid for all modalities, energy spectra and oxygen levels. The framework is based on predicting the difference in DNA-damage resulting from ionising radiation at variable oxygenation levels. *Methods.* Oxygen fixation is treated as a statistical process in a simplified model of complex and simple damage. We show that a linear transformation of the microscopic oxygen fixation process allows to extend this to all energies and modalities, resulting in a relatively simple rational polynomial expression. The model is expanded such that it can be applied for polyenergetic beams. The methodology is validated using Microdosimetric Monte Carlo Damage Simulation code (MCDS). This serves as a bootstrap to determine relevant parameters in the analytical expression, as MCDS is shown to be extensively verified with published empirical data. Double-strand break induction as calculated by this methodology is compared to published proton experiments. Finally, an example is worked out where the oxygen enhancement ratio (OER) is calculated at different positions in a clinically relevant spread out Bragg peak (SOBP) dose deposition in water. This dose deposition is obtained using a general Monte Carlo code (FLUKA) to determine dose deposition and locate fluence spectra. *Results.* For all modalities (electrons, protons), the damage categorised as complex could be parameterised to within 0.3% of the value calculated using microdosimetric Monte Carlo. The proton beam implementation showed some variation in OERs which differed slightly depending on where the assessment was made; before the SOBP, mid-SOBP or at the distal edge. Environment oxygenation was seen to be the more important variable. *Conclusions.* An analytic expression calculating complex damage depending on modality, energy spectrum, and oxygenation levels was shown to be effective and can be readily incorporated in treatment planning software, to take into account the impact of variable oxygenation, forming a first step to an optimised treatment based on biological factors.

## Introduction

The presence of oxygen during the irradiation of living tissue is known to play an important role in enhancing the biological effectiveness of dose deposition by ionising radiation (Howard-Flanders and Moore 1958, Becker and Sevilla 1993). Most effectively, when using a low linear energy transfer (LET) modality such as photons, electrons, or protons. In clinical practise, this observation leads to differences in effectiveness where hypoxic tissue acts as if it has acquired a radiation resistance and has clinically relevant consequences (Okunieff *et al* 1993). In radiation therapy with heavy charged particles, the oxygen enhancement ratio (OER) plays an important role (Jones and O’Neill 1991, Wambersie *et al* 2010). This includes the use of neutrons which deposits most of their dose through the generation of high-LET particles. It is well established that high LET particles exhibit a lower OER.

The mechanism behind this enhancement is likely a combination of physico-chemical and biological factors. A widely accepted model uses the concept of oxygen fixation, where the presence of oxygen ‘fixes’ damage in competition with chemical repair processes. Following irradiation, DNA can react with hydroxyl radicals produced nearby in the surrounding water, ultimately producing a DNA radical (DNA•). In the absence of oxygen these DNA radicals are typically restored to their original undamaged form as a result of reaction with reducing species such as thiols. However, if oxygen is present it can react with the DNA radical to produce a non-restorable organic peroxide (DNA-O_2_•) and ultimately DNA-OOH (Gray *et al* 1958, Becker and Sevilla 1993, Hall and Giaccia 2019). Experimental data demonstrate that these reactions take place on the millisecond timescale (Michael *et al* 1973, Watts *et al* 1978).

It is the goal of this work to incorporate the oxygen effect in treatment planning software. Although there are other models put forward to explain oxygenation effects, this is the most quantifiable one, resulting in quantitative predictions which can be experimentally validated.

In most models used for treatment planning, hypoxia in a targeted volume is modelled as a binary factor. Either the tissue is hypoxic or it is considered well-oxygenated. This works well in a clinical setting, indicating that the dose response to oxygenation exhibits a steep relationship. This fact has been used in a painting-by-numbers approach in the past (Van den Heuvel *et al* 2013, Madani *et al* 2015).

Other models use the observation that high-LET damage favours direct damage induction, a mechanism that does not exhibit an oxygen dependency. Therefore, a good correlation can be found between LET and the presence of oxygen effects (Grimes and Partridge 2015, Grimes 2020). Efforts within the formalism of the linear quadratic model have been worked out by other researchers (Antonovic *et al* 2014).

### Empirical model

Stewart and colleagues model the oxygen fixation hypothesis by calculating the fraction of initial DNA radicals removed through a chemical repair process (for instance, repair by thiols), which is considered a competitive channel with respect to oxygen fixation (Stewart *et al* 2011, Grimes and Partridge 2015).

We paraphrase their train of thought below:

The fraction of chemically repaired DNA radicals ${p}_{R}(y,[{{\mathrm{O}}}_{2}])$ is estimated by:

\begin{eqnarray*}{p}_{R}(y,[{{\mathrm{O}}}_{2}])=1-\displaystyle \frac{[{{\mathrm{O}}}_{2}]+K}{[{{\mathrm{O}}}_{2}]+M(y)K},\end{eqnarray*}

where $[{{\mathrm{O}}}_{2}]$ denotes the oxygen concentration (in percentage with 100% being pure oxygen). The parameter *K* is the oxygen concentration at which half of the maximum of possible repaired DNA radicals are removed, and *M*(*y*) is a function of $y\equiv {\left({z}_{\mathrm{eff}}/\beta \right)}^{2}$, the square of the ratio of effective charge of the particle to it’s speed in units of the speed of light (*c*). The function *M*(*y*) is provided by an empirical function:

\begin{eqnarray*}M(y)={M}_{0}-\displaystyle \frac{({M}_{0}-1)}{1+{\left(q/y\right)}^{r}},\end{eqnarray*}

where *M*_0_, *q*, and *r* are adjustable parameters. More specifically, *M*_0_ is the maximum fraction of DNA-radicals that can be removed through chemical repair. It is interesting to note that for very high energies the term (*q*/*y*)^*r*^ tends to a constant. This is because if $E\to \infty $ then $\beta \to 1$ and therefore:

\begin{eqnarray*}{M}_{\infty }:= \mathop{\mathrm{lim}}\limits_{E\to \infty }M(y)=\displaystyle \frac{1+{M}_{0}{\left(\tfrac{q}{{z}_{\mathrm{eff}}^{2}}\right)}^{r}}{1+{\left(\tfrac{q}{{z}_{\mathrm{eff}}^{2}}\right)}^{r}}.\end{eqnarray*}

For very low energies, equation (2) reduces to unity (as ${\left(q/y\right)}^{r}$ will tend to zero). As a consequence, equation (1) reduces to zero. In summary:

\begin{eqnarray*}\mathop{\mathrm{lim}}\limits_{\beta \to 0}{p}_{R}(y,[{{\mathrm{O}}}_{2}])=0\end{eqnarray*}

\begin{eqnarray*}\mathop{\mathrm{lim}}\limits_{\beta \to 1}{p}_{R}(y,[{{\mathrm{O}}}_{2}])=1-\displaystyle \frac{[{{\mathrm{O}}}_{2}]+K}{[{{\mathrm{O}}}_{2}]+{M}_{\infty }K}.\end{eqnarray*}

This nicely reflects the current canonic interpretation where high LET irradiation (i.e. low energy charged particles) does not exhibit oxygen enhancement of damage, and a distinct relationship with oxygen concentration at higher energies (i.e. low LET).

Alternatively, we can use the expression developed by Grimes and Partridge (2015), which is more satisfying on a physical level as it uses a specific mechanism rather than an empirical model, but we will use the first approach for reasons which will become clear below.

In this work we will rely less on a preconceived mechanism, but rather on the simpler concept of repairable and unrepairable lesions and how oxygen can alter the ratio between them.

## Methods and materials

### Set theory model

When irradiating cells with ionising radiation, DNA damage is generated through direct and indirect damage events. These damage lesions are then subjected to chemical repair in the first few milliseconds. In a longer time frame, these lesions are subjected to biological repair mechanisms.

In most models of cell death and apoptosis, a distinction is made between simple and complex damage.

(i) Simple DNA-damage lesions include single strand breaks (SSB), single base damage, and dual SSB with a spatial distance larger than a single turn of the *α-*helix (2SSB), which is roughly 10 base-pairs.
(ii) Complex damage is generally used in connection with double-strand breaks (DSB), which are split into simple DSB (just 2 strand breaks, on apposite strands) or complex DSB (simple DSB plus additional strand breaks or base damage within 10 bp). We denote this combination of damage as DSB_c_.

It is widely assumed that the latter type of damage is closely related to cell death (Ward 1985, Caldecott 2008).

In this model, we revisit the notion of oxygen fixation competing with chemical repair. First consider a system in complete hypoxia, only subjected to chemical repair. Chemical repair not only reduces the number of damage clusters which can be classified as complex damage, but can also repair simple damage. Clusters exist that are not reduced as they are already too complex and chemical repair does not affect their status. In summary, the chemical repair mechanism can convert some complex damage to simple damage, make simple damage more simple, or even completely repair them, and finally, if the damage is too extensive, fail to alter the damage. It stands to reason to differentiate between both types of damage, both of which will still be subject to other forms of repair. More specifically, the group we call complex lesions is subject to the enzymatic repair mechanisms associated with DSBs.

**Figure 1.**
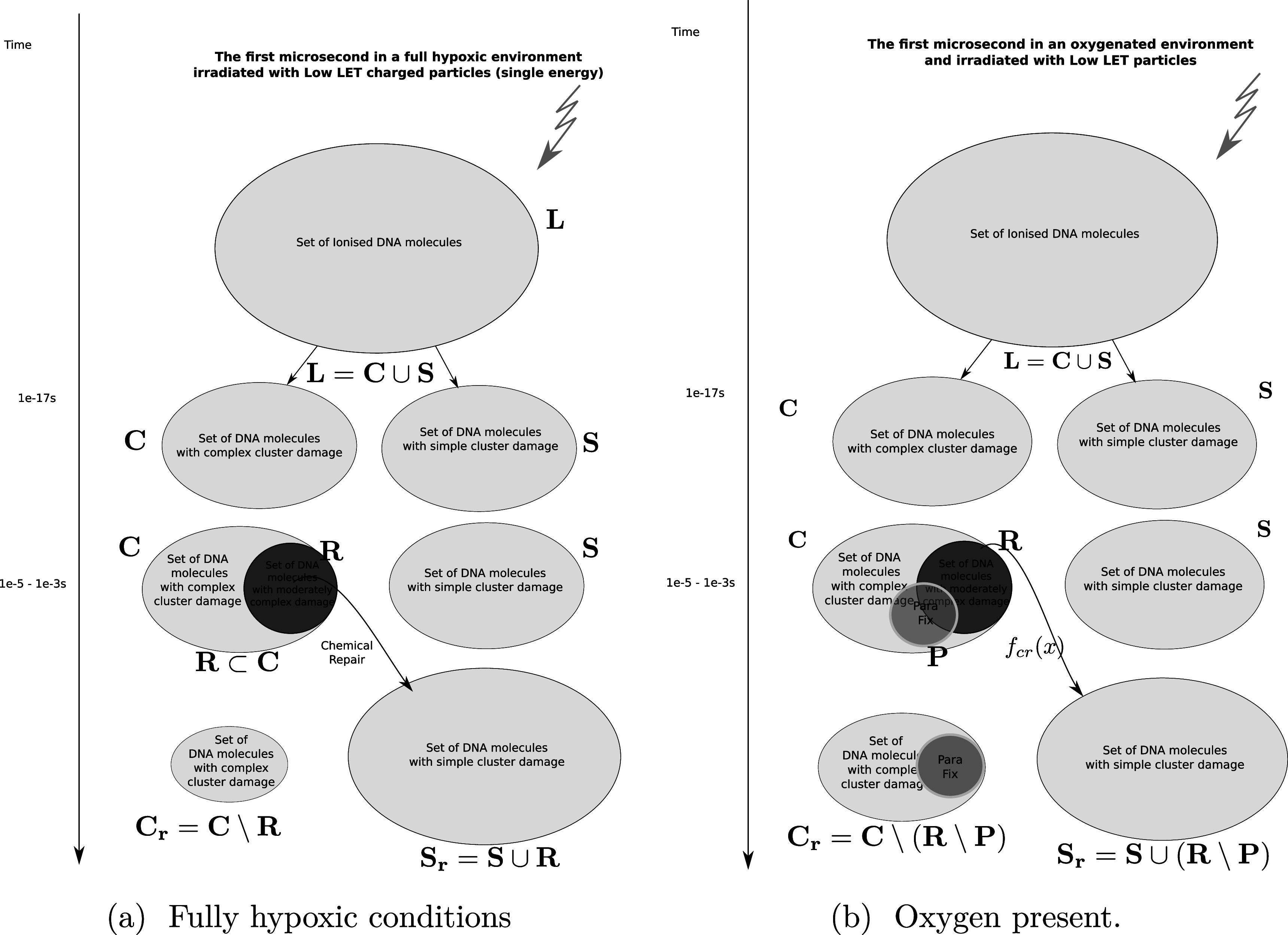
Timeflow diagram of the first microsecond of the DNA-damage process with and without the presence of oxygen.

We formalise this train of thought in terms of naive set theory (that is, no sets are considered which have an infinite number of elements) which can be graphically represented as in figure 1.

In this approach, we now define the following sets:

1. ${\bf{L}}$: The Set of all DNA-damage lesions in a cell due to a dose of 1 Gy in a cell over a length of 1 giga-base pair (Gbp).
2. ${\bf{C}}$: A subset of ${\bf{L}}$ consisting of lesions categorised as complex (including simple and complex DSB or in our notation DSB_c_).
3. ${\bf{S}}$: A subset of ${\bf{L}}$ consisting of lesions categorised as simple.
4. *f*_*cr*_: A function from ${\bf{C}}$ to ${\bf{S}}$ is used to model chemical repair. This function only works on lesions that can be repaired, in essence defining its own domain ${\bf{R}}$.
5. ${\bf{R}}$: A subset of ${\bf{C}}$ with lesions that are *repairable* using the chemical repair process.
6. ${{\bf{S}}}_{R}$: The Set of simple lesions after chemical repair.
7. ${{\bf{C}}}_{R}$: The Set of complex lesions after chemical repair.

Having defined the sets, we can now detail a number of relationships between the sets using the standard set theory symbolism: ∪ denoting the union of two sets, ∩ denoting the intersection, and \the subtraction of one set from another (i.e, it removes the common elements from the first set). Finally, we use the standard octothorpe # to denote the cardinal number of a set (i.e. the number of elements in the set).

Using these defined sets and the function, we can start quantifying the repair process.

(i) The set of complex lesions is reduced by the chemical repair process. Indeed after applying our defined function we obtain: ${f}_{{cr}}({\bf{C}})={\bf{C}}\setminus {\bf{R}}$. $\#{{\bf{C}}}_{R}$ is the amount of complex lesions left for the biological process of DNA-damage to repair. This is : $\#{{\bf{C}}}_{R}\,=\,\#{\bf{C}}-\#{\bf{R}}$, which is equivalent to the term *M*_0_ in the classical formalism.
(ii) Likewise, the set of simple lesions increases as successful chemical repair of some lesions effectively changes the categorisation of some damages from complex to simple. Indeed, ${f}_{{cr}}({\bf{C}})={\bf{S}}\cup {\bf{R}}$. In other words the function *f*_*cr*_ applied to the domain ${\bf{R}}$ is an injection in ${{\bf{S}}}_{R}$. Where ${{\bf{S}}}_{R}={\bf{S}}\cup {\bf{R}}$ or ${\bf{R}}\mathop{\longrightarrow }\limits^{{f}_{{cr}}}{{\bf{S}}}_{R}$.
(iii) By categorising the repair process as represented by the function *f*_*cr*_ as an injection over ${\bf{R}}$ we are making the assumption that all repairable lesions are indeed repaired, or that the compounds needed for the repair are not a scarce resource.

### Oxygen fixation

We introduce oxygen in the above model by using the oxygen fixation mechanism. This process makes the lesions permanent by binding the very reactive oxygen to damaged molecules (Stewart *et al* 2011). The graphical representation of this process is shown in figure 1(b). We do not distinguish which types of lesions are fixated, including lesions which are not subject to chemical repair. We keep all sets defined in the previous section and add a new one:

1. ${\bf{P}}$: A subset of ${\bf{C}}$ with lesions that have been fixated by the available oxygen.

Of interest to this model is the intersection: ${\bf{R}}\cap {\bf{P}}$, which is made up of repairable lesions that are fixated. This makes them not subject to the chemical repair process anymore. We formalise the process of chemical repair in the presence of oxygen as an injection (in mathematical terms) denoted *f*_*cr*_(*x*) with $x\in {\bf{R}}\setminus ({\bf{R}}\cap {\bf{P}})$

\begin{eqnarray*}{\bf{R}}\setminus ({\bf{R}}\cap {\bf{P}})\mathop{\longrightarrow }\limits^{{f}_{{cr}}}{{\bf{S}}}_{R}.\end{eqnarray*}

In other words, only elements of the set of repairable lesions that do not belong to the fixated lesions, are eligible for chemical repair. The resulting lesions are classified as belonging to the set of simple lesions, which is now denoted as ${{\bf{S}}}_{R}$. Conversely, ${{\bf{C}}}_{R}$ is the resulting set of complex lesions is changed accordingly as now not the total set ${\bf{R}}$ is subtracted but rather the set ${\bf{R}}\setminus {\bf{P}}$, presenting the biological repair process with $\#{{\bf{C}}}_{R}\,=\,\#{\bf{C}}-\#({\bf{R}}\setminus {\bf{P}})$ as the number of complex lesions in need of biological repair. Indeed, if there is no oxygen, then $\#{\bf{P}}=0$ and $\#{{\bf{C}}}_{R}$ is minimised. In the presence of oxygen, we assume that the original number of chemical repairable lesions does not change if we have the same modality (and energy). The only thing that changes is the number of fixated lesions which again is distributed throughout the lesions without specific preference (the set denoted as ‘parafix’)^5^ 
^5^
Short for the French: Part a fixé., making the ratio constant for a given modality and energy. Therefore, the increase of available complex lesions in need of repair is proportional to $\#{\bf{P}}$. More formally, with *a* an arbitrary constant:

\begin{eqnarray*}\#{{\bf{C}}}_{R}\,=\,\#({\bf{C}}\setminus {\bf{R}})+a.\#{\bf{P}}.\end{eqnarray*}

This implies that we need to estimate the relative number of chemically repairable lesions which are fixated. This is a ratio of the total number of fixated lesions which is given by $\#{\bf{P}}$.

In this model, we are agnostic to the specific mechanism apart from the fact that oxygen binding occurs and that oxygen is a scarce resource. This can be described mathematically by a typical pharmacological differential equation which has been generalised by Kepner as a saturation behaviour (Kepner 2010). The solution for such an equation is a two–parameter rational function of the form:

\begin{eqnarray*}\#{\bf{P}}\,=\,\displaystyle \frac{x}{{q}_{1}x+{q}_{2}}.\end{eqnarray*}

With *x* being a universal variable representing the scarce resource; in this case a good candidate is the partial oxygen pressure [O_2_] which predicts the amount of oxygen in mMOl. In expression (8), 1/*q*_1_ denotes the saturation level and 1/*q*_2_ the initial slope. The values of these parameters can then be found through the standard methodology of determining the number of oxygen molecules in a tissue related to the partial oxygen pressure.

Finally, changing our viewpoint to consider the anoxic case, the reference case, rather than the normoxic environment, the expression proposed by Stewart *et al* (equation (1)) simplifies and is equivalent to equation (8). The latter can be transformed linearly to reflect the amount of chemically repairable damage clusters. The expression in equation (7) is then re-written, using $a,b\in {\mathbb{R}}$, as:

\begin{eqnarray*}a\displaystyle \frac{x}{{q}_{1}+{q}_{2}x}+b.\end{eqnarray*}

The parameter *b* is then a measure for the amount of residual damage clusters in fully hypoxic conditions which can be reduced to three parameters,

\begin{eqnarray*}\displaystyle \frac{{{xC}}_{1}+{C}_{2}}{x+{C}_{3}}\mathrm{with}:\,{C}_{1}=\displaystyle \frac{a}{{q}_{2}}+b,\,{C}_{2}=b\displaystyle \frac{{q}_{1}}{{q}_{2}},\,{C}_{3}=\displaystyle \frac{{q}_{1}}{{q}_{2}}.\end{eqnarray*}

### Energy dependence of the oxygen effect

In earlier work, we have shown that we could describe the generation of complex damage as a function of kinetic energy of a charged particle (Van den Heuvel 2014). In this approach, we define complex damage as any damage at least as complex as a DSB. For convenience, we use the notation DSB_c_ to indicate this. In the work mentioned above, we showed that the amount of complex damage as a function of the kinetic energy *E* of a given charged particle modality can be described by an expression of the form.

\begin{eqnarray*}{{\mathrm{DSB}}}_{c}\,=\,({{dsb}}_{0}-{{dsb}}_{\infty })\left[\displaystyle \frac{1}{\pi }\arctan \left(\displaystyle \frac{{E}_{0}-E}{{\mathrm{\Gamma }}}\right)+\displaystyle \frac{1}{2}\right]+{{dsb}}_{\infty }.\end{eqnarray*}

*E*_0_ is the energy at which half of the particles interact in a high LET fashion (i.e. generating complex damage by the interaction of a single particle with a target). Γ is the width of the transition. The terms ${{dsb}}_{\infty }$ and *dsb*_0_ do not necessarily have a physical meaning. They represent the value of the inverse tangent limits. However, both ${{dsb}}_{\infty }$ and *dsb*_0_ are closely related to $\#{{\bf{C}}}_{R}$.

The expression in equation (10) is valid for all levels of available damage clusters. It needs to be adapted to the relative amount of damage for which these processes (i.e. chemical repair and oxygen fixation) can be applied. In other words, it is rescaled by $\#({\bf{R}}\setminus ({\bf{P}}\cap {\bf{R}}))$ relative to the total number of complex lesions (i.e. $\#{\bf{C}}$). In the case of high-LET charged particle interactions, we observe that $\#{\bf{C}}\gg \#({\bf{R}}\setminus ({\bf{P}}\cap {\bf{R}}))$, making any contribution of an oxygenation effect negligible. This is due to two mechanisms:

Firstly, the relative reduction of repairable lesions

\begin{eqnarray*}(\#{\bf{R}}/\#{\bf{C}})\downarrow .\end{eqnarray*}

Secondly, the relative decrease of available oxygen as it is used up by the increased number of already unrepairable lesions

\begin{eqnarray*}\displaystyle \frac{\#{\bf{P}}}{\#({\bf{C}}\setminus {\bf{R}})}\downarrow .\end{eqnarray*}

The expression in equation (11) can then be viewed as an estimate of the fraction of simpler damage (i.e. subject to chemical repair and oxygen fixation) and more complex damage.

Without loss of generality, we can assume that energy and pressure are in no way correlated. This implies that the dependence on oxygen pressure is only expressed in the parameters ${{dsb}}_{\infty }$, *dsb*_0_, *E*_0_, and Γ in equation (11). Finally, we propose that a function to describe complex damage as a function of both energy and pressure (Van den Heuvel *et al* 2013) can have the following form:

\begin{eqnarray*}{F}_{d}(E,p)=\left|{g}_{4}(p)-{g}_{3}(p)\right|\left[\displaystyle \frac{1}{\pi }\arctan \left(\displaystyle \frac{{g}_{1}(p)-E}{{g}_{2}(p)}\right)+\displaystyle \frac{1}{2}\right]+{g}_{3}(p).\end{eqnarray*}

With,

\begin{eqnarray*}{g}_{i}(x)=\displaystyle \frac{x\,{{\bf{a}}}_{i}+{{\bf{b}}}_{i}}{x+{{\bf{c}}}_{i}}\,\mathrm{for}\,i\in \{1,2,3,4\}.\end{eqnarray*}

Note that this equation equation (12) does not reflect a mechanism, but is an analytical shorthand combining the properties of both oxygen dependence and energy dependence. This expression is not derived in a mathematical sense!

To determine the parameters we will use micro dosimetric monte carlo codes to provide an estimate.

### Applying model in spectral beams

An interesting quantity to have in a treatment planning system is the a three-dimensonal map of the induced DNA-damage. Let ${\bf{D}}=D[i,j,k]$ be the dose matrix provided as calculated by a classical treatment planning system. Let ${\bf{P}}^{\prime} =p[i,j,k]$ a three-dimensional matrix representing the oxygen concentration. There exists a mapping T which transforms the spatial coordinates of ${\bf{P}}^{\prime} $ to match those of ${\bf{D}}$.

We define a new three-dimensional matrix ${{\bf{M}}}_{d}={M}_{d}[i,j,k]$ representing the damage per cell per giga-base pair (Gbp) in every voxel. For a given spectrum of a single charged particle, the dose delivered by particles with energy *E* equals the flux *ψ*(*E*) times the mass stopping power of the material of in the voxel *S*/*ρ*(*E*). Equation (12) provides the induced damage per cell, per Gbp, per Gy. Hence:

\begin{eqnarray*}{{\bf{M}}}_{{\bf{d}}}={\int }_{{E}_{0}}^{{E}_{\max }}{{\bf{F}}}_{{\bf{d}}}(E,{\bf{P}}){\boldsymbol{\psi }}(E)\displaystyle \frac{{\bf{S}}}{\rho }(E){dE}.\end{eqnarray*}

Equation (13) can therefore be used to implement a damage scoring tally in a Monte Carlo based simulation system. This is equivalent to using the energy binned F6 tally in MCNP (Stewart *et al* 2015).

In the special case of electron deposition, we note that the damage induction response is virtually independent of the energy of the electrons. Only at very low energies a relative increase in damage is observed, which is commensurate with the observed data (Hill 2004). Thus, we can apply this formalism by using dose distributions generated by off-the-shelf treatment planning systems, replacing the energy spectrum by the median energy. At clinically relevant energies, the photon dose deposition can be reduced to the dose deposition by secondary electrons, implementing it in both photon and electron treatments. Let ${\bf{P}}^{\prime} =T({\bf{P}})$, with ◦denoting the Hadamard or elementwise product and ${\bf{D}}$ the dose deposition matrix (in Gy). Then we can use the fixed median energy in equation (13) to get an estimate of the damage.

Therefore:

\begin{eqnarray*}{{\bf{M}}}_{{\bf{d}}}={\bf{D}}\circ {F}_{d}({\bf{P}},{E}_{m}).\end{eqnarray*}

Dose deposition spectra are by definition not mono-energetic and do not consist of single modalities. For any radiation source with a given energy spectrum, an energy depositing charged particle field exists in every voxel. Using general purpose Monte Carlo simulations it is possible to calculate this field and its dose deposition spectrum Ψ(*E*) in every voxel.

### Micro dosimetric Monte Carlo simulations

The use of microdosimetric calculations has provided important insight into the mechanisms and effects of radiation deposition. In the past, Monte Carlo simulations of charged particle deposition by various modalities were used to quantify and typify the kinds of damage introduced by the different modalities and many programs are available (Geant4-DNA^6^ 
^6^

http://geant4-dna.org/., Topas-nBio^7^ 
^7^

https://gray.mgh.harvard.edu/research/software/258-topas-nbio., and many others (Chatzipapas *et al* 2020)). Not forgetting the more seminal work by Nikjoo and Friedland (PARTRAC) (Nikjoo *et al* 1997, Friedland *et al* 2019).

Of specific note is the Monte Carlo Damage Simulation code (MCDS) developed by Semenenko and Stewart, which generates spatial maps of damaged nucleotides forming many types of clustered DNA lesions, including SSB, DSB, and individual or clustered base damages (Semenenko and Stewart 2006). This approach has been shown to yield a linear relationship of the number of generated DSB’s up to a high dosage. It is also the only microdosimetric simulation software that allows the inclusion of oxygen concentration in its input parameters as indicated in the introduction. MCDS version 3.10 was used with parameters as follows. The DNA length was chosen to be 1 Gbp (giga base pair) and nucleus diameter 5 *μ*m. For a more in-depth treatment of these parameters, we refer to the work by Semenenko and Stewart (2005). Variable input parameters in MCDS were; modality (i.e. energy depositing particles (electron, proton,)), energy (in MeV), and oxygen concentration in mmHg partial pressure (Torr). It is this parameter which we now allow to vary along with the energy.

The fitting procedures were performed in the gnuplot-software using a Levenberg–Marquardt minimization routine.^8^ 
^8^

http://gnuplot.sourceforge.net/.


We repeated the simulation experiment for 4 different modalities: electrons (e^−^), protons (p^+^), Helium ions ${\alpha }^{++}$, and carbon ions (${{\mathrm{C}}}^{6+}$). Here we only present electron and proton data. The kinetic energy range is presented using a logarithmic scale and such that both the high- and low-LET energy spectrum for the given modality is adequately covered. Oxygen levels vary between 0 and 100 Torr.

### Monte Carlo simulation

Equation (13) implies that to calculate the DNA-damage introduced by a clinical beam, we need to know the spectrum of the dose depositing particles. Not only for the particles in the primary beam but also for all secondary charged particles, which have their own dependency on energy and partial oxygen pressure. Having developed a validated representation of a clinical proton facility, we opted to extend the functionality of FLUKA (version 2020.0.3) to illustrate the effects of oxygen in a clinical application (Battistoni *et al* 2007, Fiorini *et al* 2018). It is clear that any general purpose Monte Carlo simulation (Agostinelli *et al* 2003, Waters *et al* 2007) or Boltzmann solver package (Vassiliev *et al* 2010) will be able to do this.

The oxygenation effect model is applied in the following scenario using the Monte Carlo simulation procedure outlined above. A combination of charged particle beams is directed at a water phantom such that a box of 10 × 10 × 10 cm^3^ receives a prescription dose. The beam is targeting a water tank of size 300 × 300 × 300 mm^3^. A spread out Bragg peak (SOBP) 10 cm long was generated using a probability distribution for the pencil beam weighting as described by Jette *et al* (Jette and Chen 2011) and Bortfeld *et al* (Bortfeld and Schlegel 1996), the maximal nominal energy of the proton pencil beams used was 180 MeV.

In FLUKA, non-standard scoring typically requires including scripts such as fluscw.f in the input file to weigh the standard fluence distribution and tally the desired scoring. USRBIN standard fluence-based scoring can be modified to tally DSB_c_ damage as follows. We determined the DSB_c_ parameters in equation (11) for electrons, protons, deuteron, helium ions, Lithium ions, and carbon ions at 0%, 0.1%, 10%, 20% and 50% oxygen levels. These were included in fluscw.f. DSB_c_ is calculated as in equation (12), using the kinetic energy of the correspondent particle. The equivalent dose (in Gy) for each particle at different oxygen concentrations is then calculated by multiplying the DSB_c_ by the stopping power through the GETLET() function in the medium (Battistoni *et al* 2007). Indeed, the function *F*_*d*_(*E*, *P*) is expressed per Gy delivered and dose is calculated as fluence times the stopping power. Similarly, the kinetic energy, particle fluence and LET are recorded separately within the same volume.

Each USRBIN scoring is defined as a cylinder of 100 mm radius, 300 mm deep. The cylinder is subdivided in slices of 1 mm thick and 100 radii, yielding annuli 1 mm thick. The number of simulated primaries is set equal to 10^8^.

The standard dose in Gy is calculated in commscw.f independently to compare the standard dose calculation and appreciate the oxygenation effects.

Finally, this is repeated for all secondary particles and added to the result in every single scoring volume.

The process is repeated in different oxygenation environments: 0%, 0.1%, 10%, and 20% pO_2_. Following the rationale defining damage RBE, a damage OER (OER_d_) for a particle with energy *E* is defined such that:

\begin{eqnarray*}{{\mathrm{OER}}}_{{\mathrm{d}}}(E,[{{\mathrm{O}}}_{2}])=\displaystyle \frac{{M}_{{\mathrm{d}}}(E,[{{\mathrm{O}}}_{2}])}{{M}_{{\mathrm{d}}}(E,0)}.\end{eqnarray*}

### Comparison with published data

In a paper by Prise *et al* (1990), mammalian V79 cells were irradiated using proton beams with relatively low energy (i.e. 0.76, 1.15, and 1.90 MeV mean energy) in both fully hypoxic and atmospheric conditions. Not only cell survival curves were determined, but also an estimate of the generated DSB was performed using a neutral filter elution technique (Bradley and Kohn 1979, Prise *et al* 1989).

The irradiation set up and specific spectral data was found in an additional paper by the same group (Folkard *et al* 1989). We resampled the proton spectra provided in this paper on a grid with a 0.01 MeV resolution. Using expression (13) at different oxygenation levels *M*_d_(*E*, *p*) (with *p* = 0%, 0.1%, 1%, 10%, and 20%) we calculated the number of DSB generated per Gy, per cell, per giga-base pairs (Gbp). Applying equation (15) we readily obtained the OER_d_ in the different oxygenation conditions. We considered 20% partial oxygenation to be the atmospheric conditions.

## Results

### Oxygen fit

**Figure 2.**
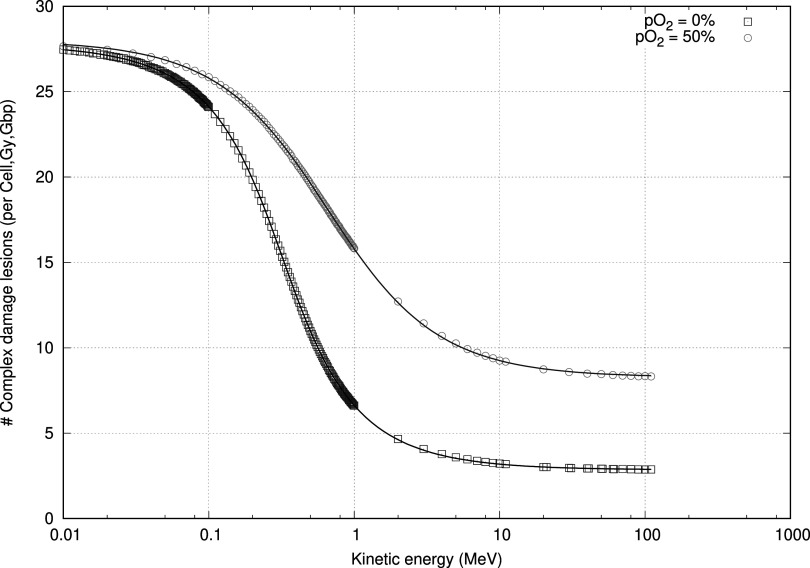
Applying equation (12), to calculate the complex damage (DSB_c_) for protons at anoxic (0% oxygen) and hyperbaric oxygen conditions 50% oxygen (380 Torr), which is indistinguishable from the 20% (152 Torr) atmospheric condition. Lines are this work. Points are from MCDS simulations.

**Figure 3.**
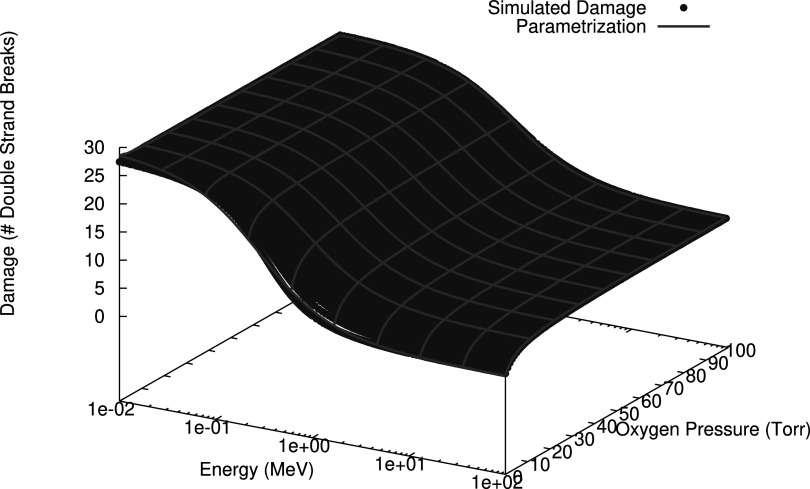
Dependency of the induction of complex lesions in DNA (defined as in the definition of DSB_c_) by protons as a function of kinetic energy and partial oxygen pressure. The lines represent the parameterisation as proposed in equation (12).

The determination of the various parameters and the resulting goodness of fit is shown in figure 2 for two different oxygenation levels calculated using equation (11). Furthermore, it is illustrated over a range as a three-dimensional plot in figure 3 which used equation (13). In table 1 we summarize the statistics of the residual errors of the latter fit. Values of the number of complex lesions range between 5 and 30 expressed per cell, per giga base pair (Gbp), and per Gy. Most outliers are found in the energy range in which the transition of low to high LET regimen occurs.

**Table 1.** Distribution of residuals. Standard deviation of the order of 1%, showing that good agreement with MCDS is found.

Modality	Energy Range	Std Dev	Range
e^−^	[1e-07–10 MeV]	0.16	[−1.18 −1.18]
p^+^	[0.01–1000 MeV]	0.21	[−0.52 −1.37]

### Oxygen effect in a proton SOBP

**Figure 4.**
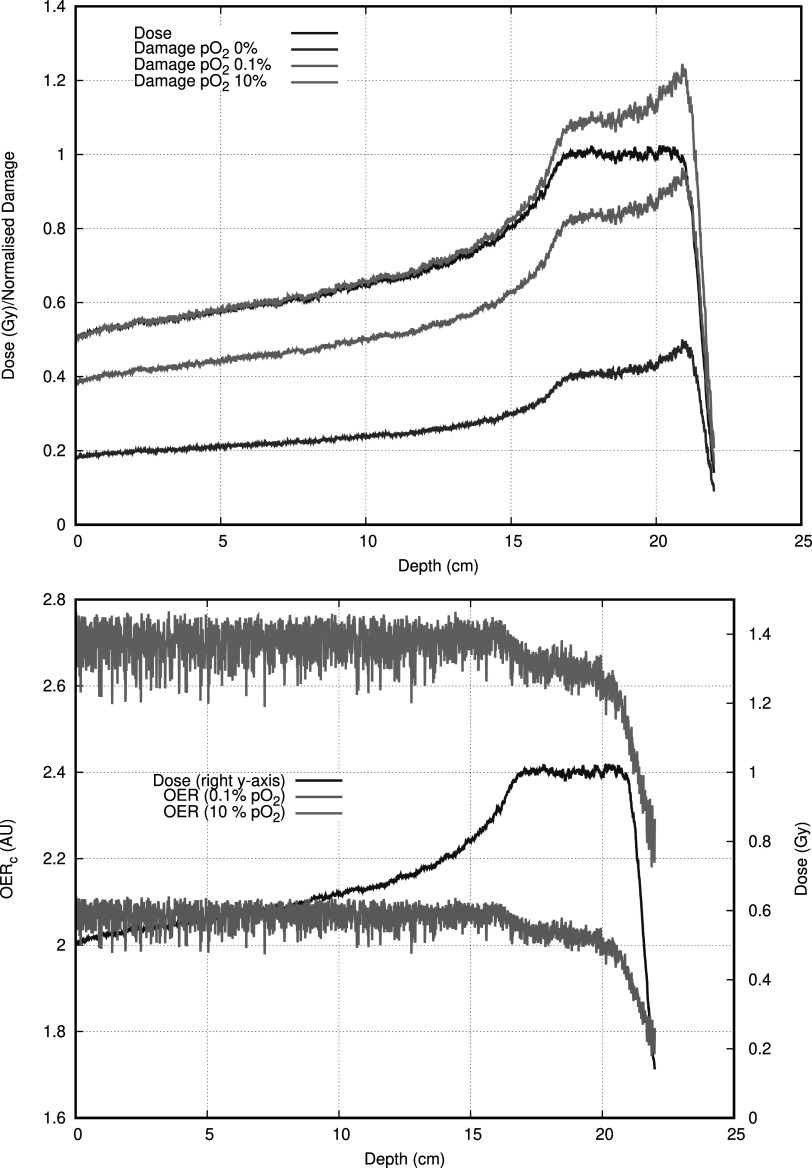
Top: the effective dose at the central axis of a 10 × 10 cm^2^ proton SOBP at different oxgygenation levels. The 10% oxygenation is normalised to the dose deposition at 0.5 cm depth. Bottom: the oxygen enhancement ratio with reference to the fully hypoxic environment. For comparison the dose deposition curve is also shown. The OER_d_ is roughly constant except at the bragg peak.

Figure 4 shows the depth dose curve of the centre of a 10 × 10 cm^2^ size proton treatment with an SOBP of 10 cm. The dose is presented, but also the curves of the damage at 10% normalised to the dose at 0.5 cm depth. As such, the damage curves can be read as an effective dose.

On the totality of the irradiation the OER_d_ increases following increased oxygenation, which is expected. Interestingly the OER_d_ decreases slightly as the SOBP is reached and roughly stays constant in the high dose volume. Towards the end and past the SOBP the OER_d_ decreases as the lower energy protons become increasingly more important in the proton spectrum. Within the high dose volume the decrease in OER_d_ is of the order of 3% percent, which is probably not important enough for clinical purpose as the variation in oxygenation combined with a variety of repair effects will drown out this effect. Once behind the bragg peak the OER_d_ reduces by about 10%, but almost no dose is deposited.

### Comparison with published data

In table 2 we compare the calculated values of OER_d_ for beams with a mean energy of 0.76 and 1.15 MeV. The values obtained at 20% partial oxygen pressure are considered equivalent to standard atmospheric environments. Prise *et al* also determined the OER_d_ for an x-ray beam with nominal energy of 250 kVp. Making some rough assumptions, namely, that all dose is deposited by secondary electrons with a median energy of 100 keV and taking advantage of the flat response of DSB induction at electron energies above 10 keV, we find a value of 2.861, while 3.50 is reported.

**Table 2.** Calculated and measured oxygen enhancement ratios based on differences in induced number of double strand breaks (OER_d_) in mammalian V97 cells irradiated using protons having different LET. This is shown at different partial oxygen pressures, with 20% being the atmospheric pressure. Measurement data are from Prise *et al* (1990), using the same methodology this group quote an OER_d_ = 3.50 for an x-ray beam at 250 kVp.

	OER_d_						
LET (keV *μ*m^−1^)	$\langle {E}\rangle $ (MeV)	0.1%	1%	10%	20%	Mean	Prise *et al*
17.0	1.90 MeV	1.810	2.226	2.271	2.293	2.344	—
24.0	1.15 MeV	1.500	1.715	1.732	1.743	1.915	**1.64**
32.0	0.76 MeV	1.317	1.441	1.451	1.457	1.555	**1.49**

## Discussion

The methodology developed above is satisfying on a physical and mathematical level in that it allows to describe the interplay between energy and oxygenation adequately in one model. In addition, there are no ‘unnatural’ discontinuities, nor assumptions based on *a priori* observations. Grimes’ approach (Grimes 2020), implicitly assumes that direct DNA damage is not subject to oxygen fixation. Grimes argues that it is the ratio between direct and indirect damage that drives the oxygenation effects. In contrast, in our model this is not the case as high LET particles indeed generate more direct damage but also generate more complex damage, in effect diminishing any contribution of oxygen fixation.

In the work presented by Stewart *et al* (2015) the oxygenation effect is calculated in part by an empirical function, which we show to be equivalent to our approach in a low LET regimen. At higher LET values, the oxygenation effect is assumed to be non-existent by definition. In this work, the effect of oxygenation gradually becomes less important as the complexity of damage increases and the impact of oxygen becomes vanishingly small, but not necessarily zero. All this using a model based on first principles.

A justified critique to this work could be that it only models the induction of DNA-damage and that enzymatic DNA-damage repair has not been taken into account. This is not entirely true, as we consider this model as the first step in a damage–repair–misrepair sequence. However, we need to be aware that this approach is only valid if there is a clear relationship of the amount of DNA-damage induced and the survival of the cell. Indeed, it is important that the individual repair–capacity of cells are taken into account when dealing with the spectrum of DNA damage complexity and the modification of this damage with level of oxygen present. For example, in the extreme of repair deficient cells, minimal increase in RBE for cell survival is observed with increasing LET. Ewing argued that therefore the mechanism of oxygen enhancement somehow impacts the repair process itself (Ewing 1998). While for other cell types, such as haemopeitic stem cells, it is known that they are very sensitive to ionising radiation and will preferentially undergo apoptosis rather than repair damage, regardless of the variability introduced by oxygen fixation. This is in addition to the observation that DNA damage repair operates on a vastly different time scale (minutes versus microseconds/milliseconds).

## Conclusions

We have introduced an alternative treatment of the concept of oxygen fixation, by considering it as a statistical process that competes to an alternative process (i.e. chemical repair). While the concept is not new it is the quantification and the gradual change of the impact as the LET of various modalities increases, which is quite novel. In addition, the concept can be extended to other particle types, the investigation of which will be presented in future work.
